# Integrated Analysis of the Transcriptome and Metabolome Revealed the Molecular Mechanisms Underlying the Enhanced Salt Tolerance of Rice Due to the Application of Exogenous Melatonin

**DOI:** 10.3389/fpls.2020.618680

**Published:** 2021-01-14

**Authors:** Ziyan Xie, Juan Wang, Wensheng Wang, Yanru Wang, Jianlong Xu, Zhikang Li, Xiuqin Zhao, Binying Fu

**Affiliations:** ^1^Institute of Crop Sciences/National Key Facility for Crop Gene Resources and Genetic Improvement, Chinese Academy of Agricultural Sciences, Beijing, China; ^2^College of Agronomy, Anhui Agricultural University, Hefei, China

**Keywords:** salinity stress, transcriptional cascade, metabolite, *Oryza sativa*, phytohormon, antioxidant

## Abstract

High salinity is one of the major abiotic stresses limiting rice production. Melatonin has been implicated in the salt tolerance of rice. However, the molecular basis of melatonin-mediated salt tolerance in rice remains unclear. In the present study, we performed an integrated transcriptome and metabolome profiling of rice seedlings treated with salt, melatonin, or salt + melatonin. The application of exogenous melatonin increased the salt tolerance of rice plants by decreasing the sodium content to maintain Na^+^/K^+^ homeostasis, alleviating membrane lipid oxidation, and enhancing chlorophyll contention. A comparative transcriptome analysis revealed that complex molecular pathways contribute to melatonin-mediated salt tolerance. More specifically, the AP2/EREBP–HB–WRKY transcriptional cascade and phytohormone (e.g., auxin and abscisic acid) signaling pathways were activated by an exogenous melatonin treatment. On the basis of metabolome profiles, 64 metabolites, such as amino acids, organic acids, nucleotides, and secondary metabolites, were identified with increased abundances only in plants treated with salt + melatonin. Several of these metabolites including endogenous melatonin and its intermediates (5-hydroxy-L-tryptophan, *N*^1^-acetyl-*N*^2^-formyl-5-methoxykynuramine), gallic acid, diosmetin, and cyanidin 3-*O*-galactoside had antioxidant functions, suggesting melatonin activates multiple antioxidant pathways to alleviate the detrimental effects of salt stress. Combined transcriptome and metabolome analyses revealed a few gene–metabolite networks related to various pathways, including linoleic acid metabolism and amino acid metabolism that are important for melatonin-mediated salt tolerance. The data presented herein may be useful for further elucidating the multiple regulatory roles of melatonin in plant responses to abiotic stresses.

## Highlights

- An exogenous melatonin treatment enhances rice salt tolerance by activating distinct transcriptional cascades and phytohormone signaling in concert with multiple antioxidants and unique metabolic pathways.

## Introduction

Melatonin is a low-molecular-weight indoleamine compound that was first discovered in the pineal gland in 1958 (Lerner et al., 1958). It was characterized as a neurohormone exclusive to animals and associated with diverse physiological systems (Reiter, 1991). In 1995, melatonin was detected in a few edible plants based on mass spectrometry combined with fluorescence detection and other techniques (Dubbels et al., 1995; Hattori et al., 1995). Subsequent research revealed that melatonin is widely distributed in the plant kingdom and has multiple biological activities (Arnao and Hernández-Ruiz, 2015). There are five types of genes involved in the melatonin biosynthesis and catabolism in plants. Specifically, genes encoding four consecutive enzymes, tryptophan decarboxylase, tryptamine 5-hydroxylase, serotonin *N*-acetyltransferase, and *N*-acetylserotonin *O*-methyltransferase, are related to melatonin biosynthesis, whereas one gene encoding melatonin 2-hydroxylase helps mediate melatonin catabolism (Arnao and Hernández-Ruiz, 2015; Wei et al., 2016). Most of these genes have been identified and characterized in plants and animals, with a few genes named differently and encoding proteins with diverse functions between plants and animals (Arnao and Hernández-Ruiz, 2014).

There is considerable evidence that melatonin has multiple regulatory effects on plant growth and development (Arnao and Hernández-Ruiz, 2014). Most importantly, melatonin is a key factor influencing the regulation of plant tolerance to biotic and abiotic stresses. For example, its strong antioxidant activity helps maintain reactive oxygen species homeostasis to protect plants from damages caused by environmental stresses (Kolář and Macháčková, 2005; Sun et al., 2020a). Melatonin can also activate various antioxidant enzymes, such as superoxide dismutase, catalase, peroxidase, and ascorbate peroxidase, to mitigate oxidative stress in plants (Shi et al., 2015b; Khan et al., 2020). Plant photosynthesis is very sensitive to environmental stresses, including extreme temperatures, drought, and salinity. Melatonin can increase chlorophyll contents as well as enhance electron transport and stomatal conductance to restrict the adverse effects of abiotic stresses on photosynthesis (Wang et al., 2013, 2018; Zhang et al., 2015). Exogenous melatonin reportedly inhibits chlorophyll degradation, suppresses the expression of senescence-associated genes, and delays leaf senescence by regulating a few transcription factors (TFs), including senescence-related SGR and NAC TFs (Liang et al., 2015). The application of exogenous melatonin can modulate the expression of endogenous genes in plants exposed to abiotic stresses. Several studies revealed that TF genes, including those encoding drought-responsive element-binding (DREB), WRKY, MYB, bHLH, NAC, and HSF TFs, are significantly regulated in plants by melatonin (Weeda et al., 2014; Shi et al., 2015a; Li et al., 2016). These melatonin-activated TF genes under abiotic stress conditions are important for regulating the downstream stress-response genes. Melatonin can alleviate the inhibitory effects of abiotic stress on gene expression, with many downstream genes related to primary and secondary metabolism differentially expressed following a melatonin pre-treatment (Shi et al., 2015a; Hu et al., 2020).

With the development of metabolome-profiling platforms, global changes to metabolites in response to a melatonin treatment have been investigated based on gas chromatography–mass spectrometry and liquid chromatography–mass spectrometry (LC-MS) analyses. Previous studies confirmed that several primary metabolites, including amino acids, organic acids, and sugars, are involved in melatonin-mediated responses to biotic and abiotic stresses (Shi et al., 2015a; Hu et al., 2016). Additionally, secondary metabolites, such as linoleic acid, flavonoids, and intermediates of melatonin biosynthesis, increase in abundance after an exogenous melatonin treatment of plants under stress conditions (Yu et al., 2018; Debnath et al., 2020; Hu et al., 2020).

Rice is one of the most sensitive cereal crops to salinity. Earlier research indicated that the application of diverse exogenous biochemical compounds, including phytohormones, growth regulators, and antioxidants, can improve rice salt tolerance (Wang et al., 2016; Ganie et al., 2019). Melatonin alleviates salinity stress in rice by detoxifying the H_2_O_2_ accumulated in cells and enhancing the activity of the plasma membrane K^+^ transporter to maintain K^+^ homeostasis (Liang et al., 2015; Liu et al., 2020). However, there is little information regarding the melatonin-mediated metabolomic and transcriptomic responses of rice plants to salt stress. To characterize the molecular mechanisms underlying the effects of melatonin more comprehensively on salt stress tolerance, we comparatively analyzed the transcriptome and metabolome of rice seedlings following control, melatonin, salt stress, and salt stress plus melatonin treatments. The combined global gene expression and metabolite profiles revealed exogenous melatonin can enhance rice salt tolerance by activating distinct transcriptional cascades and unique metabolic pathways. The results of this study provide novel insights into the complex regulatory activities underlying melatonin-mediated salt stress tolerance in rice.

## Materials and Methods

### Plant Growth and Treatments

A highly salt-sensitive *Oryza sativa* L. ssp. *Geng* (*japonica*) rice variety, 02428, was used as the study material. The seeds were surface-sterilized with 4% sodium hypochlorite for 20 min, rinsed with distilled water, and then germinated in an incubator. The germinated seeds were placed in the wells (containing tap water) of 96-well PCR plates and incubated for 7 days, after which they were transferred to Yoshida nutrient solution (Yoshida et al., 1976). The rice seedlings at the 3-leaf stage were independently subjected to the following four treatments, with five replicates per treatment: control (normal nutrient solution), salt (nutrient solution + 100 mM NaCl), melatonin (nutrient solution+10 μM melatonin), and salt + melatonin (nutrient solution + 100 mM NaCl + 10 μM melatonin). The seedlings were grown in a phytotron (size: 4.0 m × 2.5 m × 2.5 m) with a day (29°C)/night (22°C) cycle, an irradiance of about 700 μmol quanta m^–2^ s^–1^, and a minimum relative humidity of 70%. The pH of the nutrient solution was adjusted daily to 5.5 by adding sulfuric acid and the nutrient solution was refreshed weekly. Leaf samples were collected at 7 days post-treatment for the transcriptome and metabolome analyses. Freshly harvested leaves were quickly frozen in liquid nitrogen and stored at −80°C until the total RNA and metabolite extractions.

### Physiological Trait Analysis

The 02428 seedlings were collected at 7 days post-treatment for physiological analysis. The leaf chlorophyll content of five plants per treatment was measured with the SPAD-502 meter (Minolta Camera Co., Ltd., Japan) according to the instruction. The chlorophyll meter reading was detected in the first fully expanded leaf, and five SPAD readings were taken around the midpoint of each leaf and averaged its values. The Na^+^ and K^+^ contents of the shoots and roots were measured at 7-days post-treatment as described by Zang et al. (2008). Briefly, shoots and roots were dried under 80?, weighed and extracted in acetic acid (100 mmol/L) at 90? for 2 h. Na^+^ and K^+^ levels in the acid-digested samples were estimated using the flame photometer (S2, Thermo Finnigan, Waltham, MA United States). The concentrations of Na^+^ and K^+^ were determined at 589 and 766.5 nm, respectively. The malondialdehyde (MDA) content was measured using thiobarbituric acid (TBA) as described by Zhang et al. (2019). Specifically, MDA was extracted using chilled TBA and quantified by determining the absorbance of the supernatant at 532 nm.

### Melatonin Extraction and Detection

The shoot melatonin concentration of the 02428 seedlings was measured based on ultra performance liquid chromatography (UPLC) as previously described (Sun et al., 2020b) with minor modification. Leaf samples were ground in liquid nitrogen and extracted with 1.5 mL methanol solution under precooled conditions. After sonication and centrifugation, the supernatant was collected and dried under nitrogen. The residue was redissolved in 2 mL of 5% methanol-water solution for further isolation of melatonin using the solid phase extraction (SPE). The C18 SPE cartridge was activated as described by Chen et al. (2003). The 2 mL prepared sample was added to the column, after drying, 10 mL of 5% methanol-water solution was used to elute the interference impurities, then retained melatonin was eluted at a low flow-rate using 1 mL of 80% methanol-water solution, the eluate was then filtered and subjected for UPLC/MS analysis.

The analytes were separated using Agilent Z0RBAX Eclipse XDB-C18column (4.6 mm × 250 mm, 5 μm) with water (solvent A) and methanol (solvent B) as mobile phases at a flow rate of 0.8 mL/min. Ten μL extracted sample was injected for the analyses. The excitation wavelength was selected at 286 nm and the emission wavelength was selected as 352 nm for melatonin detection using fluorescence.

### RNA Extraction, Library Construction, RNA Sequencing, and Data Analysis

Total RNA was extracted from frozen leaf samples using the TRIzol reagent, after which it was purified and concentrated with the RNeasy MinElute Cleanup kit (Qiagen). For each sample, 1 μg RNA was used as the input material for preparing sequencing libraries using the NEBNext Ultra^TM^ RNA Library Prep Kit for Illumina (NEB, United States). Index codes were added to attribute sequences to each sample. The median insert size was set at 200–300 bp. The prepared libraries were sequenced with the Illumina HiSeq 4000 system, with three biological replicates per sample. The raw sequence data have been deposited in the Genome Sequence Archive in National Genomics Data Center, Beijing Institute of Genomics, Chinese Academy of Sciences, under accession number CRA003581 that are publicly accessible at^1^.

Raw reads in the fastq format were first processed with in-house perl scripts. Clean reads were obtained by removing reads containing adapters, reads with poly-*N* sequences, and low-quality reads from the raw data. The clean reads were then mapped to the reference genome sequence using HISAT2. Gene expression levels were estimated based on the fragments per kilobase of transcript per million fragments mapped. The genes differentially expressed between the treated and control samples (false discovery rate < 0.01 and fold-change ≥2) were identified using EBseq (Leng and Kendziorski, 2020).

A Gene Ontology (GO) enrichment analysis of the differentially expressed genes (DEGs) was performed using the agriGO toolkit^2^ Tian et al., 2017). The KOBAS software (Xie et al., 2011) was used to identify the Kyoto Encyclopedia of Genes and Genomes (KEGG) pathways enriched among the DEGs. A quantitative real-time PCR (RT-qPCR) analysis was performed to confirm the expression levels of selected genes involved in melatonin biosynthesis and catabolism. Details regarding the analyzed genes and the gene-specific primers are listed in Supplementary Table 1.

### Metabolite Profiling and Data Analysis

Metabolite profiles were determined using the same materials that were used for the transcriptome analysis. Metabolites were extracted from 12 leaf samples (i.e., three biological replicates for each of the four treatments). The frozen samples were freeze-dried and ground for 1.5 min at 30 Hz using the MM 400 mixer mill (Tetsch). Metabolites were extracted by adding 1.0 mL 70% aqueous methanol to 100 mg ground material and incubating overnight at 4°C. Samples were centrifuged at 10,000 × *g* for 10 min, after which the extracts were filtered with the SCAA-104 filter (0.22 μm pore size) prior to the LC-MS analysis.

The metabolite profiling was completed using a platform that combined UPLC and tandem mass spectrometry (MS/MS) as previously described (Guo et al., 2019). Metabolites were characterized using the Metware database (Metware Biotechnology Co., Ltd., Wuhan). Additionally, they were quantified based on the peak areas with the multiple reaction monitoring mode. The metabolite data have been deposited in the National Genomics Data Center, Beijing Institute of Genomics, Chinese Academy of Sciences, under accession number OMIX220 that are publicly accessible at^3^.

The metabolite profiling data were normalized for a principal component analysis (PCA) (Jolliffe, 2002). The metabolites that were differentially abundant between the control and treated 02428 seedlings were detected based on the following criteria: variable importance in projection (VIP) ≥1 and a fold-change ≥1.5. The significantly enriched KEGG pathways were identified with Fisher’s exact test using a FDR < 0.05.

### Correlation Analysis of Metabolite and Transcript Profiles

The metabolite and transcript data were converted to log_2_ values for subsequent analyses. Pearson correlation coefficients (PCCs) were used to analyze the metabolites and transcripts for the combined metabolome and transcriptome analysis. Correlations were determined based on the following criteria: PCC > 0.80 and corresponding *p*-value < 0.05.

## Results

### Melatonin Improved the Physiological Traits of Rice Seedlings Under Salt Stress Conditions

**FIGURE 1:**
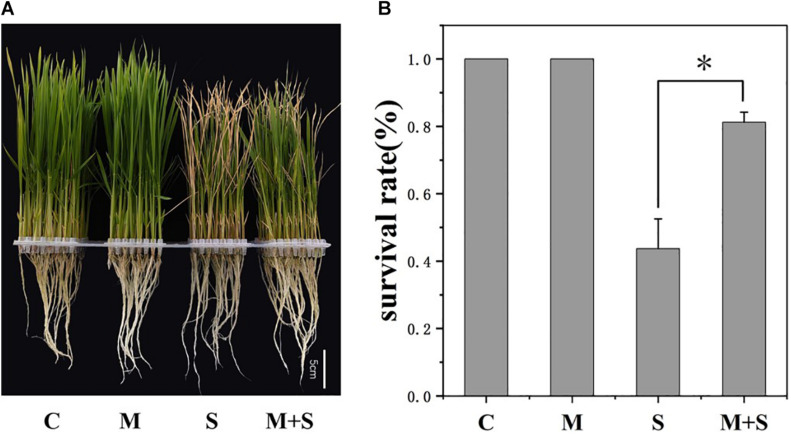
Melatonin effects on the 02428 seedlings under salt stress conditions. **(A)** Phenotypes of the 02428 seedlings after different treatments. **(B)** Survival rates of the treated seedlings after a 5-day recovery period. Data were collected from three independent replicates, and values are presented as the mean ± standard deviation of 30 measurements. *p ≤ 0.05 (Student’s *t*-test). C, control; M, 10 μM melatonin; S, 100 mM NaCl; M+S, 100 mM NaCl + 10 μM melatonin.

**FIGURE 2:**
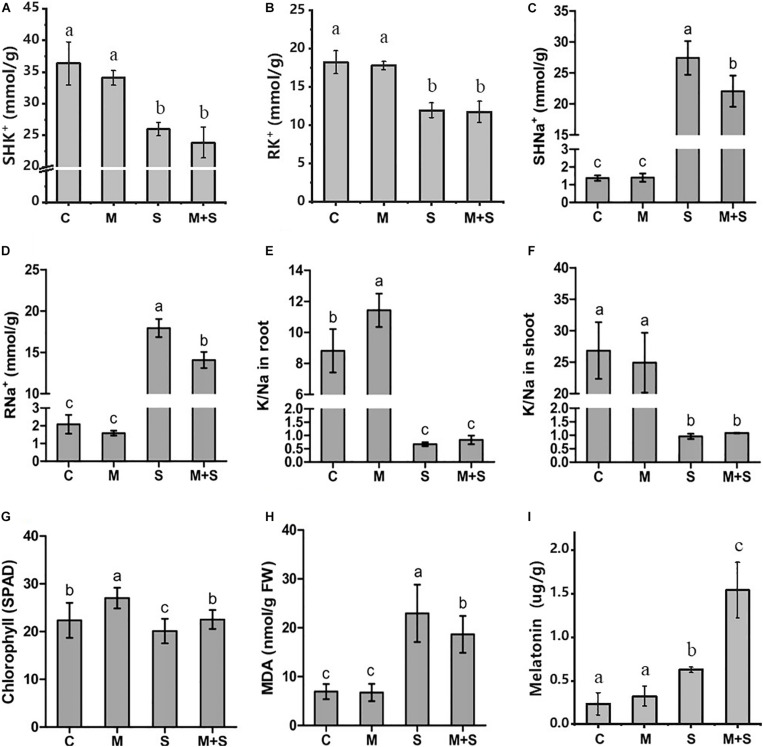
Physiological traits of the 02428 seedlings after different treatments. C, control; M, 10 μM melatonin treatment; S, 100 mM NaCl treatment; M+S, 10 μM melatonin + 100 mM NaCl treatment; SH, shoots; R, roots; Na+, sodium concentration; K+, potassium concentration; K/Na, ratio of potassium to sodium; MDA, malondialdehyde. Data are presented as the mean ± standard error (*n* = 5). Different letters above the columns indicate significant differences between treatments at *p* < 0.05 (Tukey’s range test).

To investigate the physiological effects of exogenous melatonin on rice plants exposed to salt stress, the 02428 seedlings at the 3-leaf stage were treated with 100 mM NaCl, 10 μM melatonin, or 100 mM NaCl + 10 μM melatonin for 7 days. There were no phenotypic differences between the melatonin-treated seedlings and the control seedlings (Figure 1). However, the exogenous melatonin alleviated the detrimental effects of salt stress, with the survival rate of the seedlings that underwent the salt + melatonin treatment (80%) significantly higher than that of the seedlings treated with salt alone (45%) (Figure 1B). Under normal growth conditions, the application of exogenous melatonin increased the K^+^/Na^+^ ratio in the roots as well as the chlorophyll contents in the shoots (Figures 2E,G). Under salt stress conditions, the exogenous melatonin mitigated the overall effect of salt stress. In contrast to the significantly increased endogenous melatonin level (Figure 2I), the sodium contents in the shoots and roots decreased in response to exogenous melatonin (Figures 2C,D). Additionally, exogenous melatonin increased the chlorophyll content, but decreased the MDA content in the shoots under salt stress conditions (Figures 2G,H). These results indicate that exogenous melatonin may offset the effects of salt stress by decreasing the sodium concentration, enhancing chlorophyll contention, and maintaining membrane stability.

### Genome-Wide Transcriptomic Responses of 02428 Seedlings to Melatonin, Salt, and Salt + Melatonin Treatments

The global transcriptional changes in rice seedlings induced by different treatment conditions were analyzed by RNA sequencing (RNA-seq) to identify the DEGs. There were only 16 DEGs (11 up-regulated, 5 down-regulated) detected in 02428 under exogenous melatonin treatment relative to control, and 189 DEGs (150 up-regulated, 39 down-regulated) identified under salt + melatonin relative to melatonin treatment (Table 1); More specifically, 3,274 (2,279 up-regulated and 995 down-regulated) and 3,995 (2,573 up-regulated and 1,422 down-regulated) genes were significantly differentially expressed in the 02428 seedlings after the salt and salt + melatonin treatments, respectively, compared with the control levels (Table 1 and Supplementary Tables 2, 3). The expression levels of 11 genes involved in melatonin biosynthesis and catabolism were determined by RT-qPCR to verify the RNA-seq data. The RT-qPCR results were consistent with the RNA-seq data (*r*^2^ = 0.79; Supplementary Figure 1 and Supplementary Table 1).

**TABLE 1:** Summary of the differentially expressed genes in the 02428 seedlings treated with salt and salt + melatonin.

Conditions	Up-regulated	Down-regulated	Sub-total
Melatonin vs control	11	5	16
Salt versus control	2,279	995	3,274
Salt + melatonin versus melatonin	150	39	189
Salt + melatonin versus control	2,573	1,422	3,995

**FIGURE 3:**
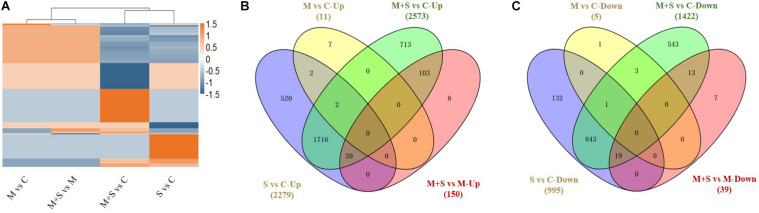
Cluster analysis of all DEGs in 02428 seedling under different treatments **(A)** and Venn diagram analysis of differentially up-regulated **(B)** and down-regulated **(C)** genes in the 02428 seedlings under different treatments. M, melatonin treatment; M+ S, melatonin + salt treatment; S, salt treatment; Up, up-regulated genes; Down, down-regulated genes. The differentially expressed genes under different treatments were identified based on the following criteria: fold-change ≥2 and FDR < 0.01.

Table 1 and Figure 3 summarize the number of up- and down-regulated genes in the seedlings under different treatments, revealing general patterns of transcriptomic responses of 02428 to melatonin, salt, and salt + melatonin. More genes were differentially regulated by salt + melatonin than by salt alone, and there were more up-regulated genes than down-regulated genes in the seedlings following the salt and salt + melatonin treatments. Cluster analysis differentiated these DEGs into two major clusters by stress effect (melatonin + salt vs control, salt vs control) and melatonin effect (melatonin vs control, melatonin + salt vs melatonin) (Figure 3A). Further Venn diagram analysis showed that the DEGs in the comparison of melatonin + salt vs melatonin were mostly included in those of the comparison of melatonin + salt vs control (Figures 3B,C). Therefore, further analyses for the melatonin-mediated molecular pathways in the following section were focused on comparative analysis of the DEGs in comparisons of salt vs control and melatonin + salt vs control.

### Exogenous Melatonin Mitigates the Salt Stress Effects on Rice Plants by Specifically Regulating Various Genes With Diverse Functions

The application of exogenous melatonin up- and down-regulated the expression of 11 and 5 genes, respectively, in the 02428 seedlings under control conditions (Table 1). The up-regulated genes included *OsEXPB2* (Os10g0555700), *Hsp40* (Os01g0927400), and several putative protein coding genes, whereas the down-regulated genes were *OsBBX20* (Os06g0654900), *OsLTP2.12* (Os10g0505700), and three protein coding genes with unknown functions. Accordingly, the exogenous melatonin only slightly affected the global gene expression in the 02428 seedlings under normal growth conditions.

The salt and salt + melatonin treatments substantially affected genome-wide gene expression. A comparison of the up- and down-regulated genes in a Venn diagram indicated that 816 and 559 genes were exclusively up- and down-regulated, respectively, in the 02428 seedlings that underwent the salt + melatonin treatment (Figures 3B,C), reflecting the considerable effect of the exogenous melatonin on the global gene expression in rice plants under saline conditions.

A GO enrichment analysis was performed for these exclusive DEGs using the agriGO online tool^4^. The following four GO categories were enriched among the up- and down-regulated genes (Supplementary Figure 2): receptor activity (GO:0004872), mitochondrion (GO:0005739), intrinsic to membrane (GO:0031224), and chloroplast (GO:0009507). The most enriched categories among the 816 up-regulated genes were developmental process (GO:0032502), response to hormone stimulus (GO:0009725), response to abiotic stimulus (GO:0009628), cell wall organization or biogenesis (GO:0071554), regulation of transcription (GO:0006355), carbohydrate metabolic process (GO:0005975), and apoplast (GO:0048046). Thus, genes encoding functionally diverse proteins contribute to melatonin-mediated rice plant responses to salt stress. The main enriched GO categories among the down-regulated genes were membrane part (GO:0044425), photosynthetic membrane (GO:0034357), signal transducer activity (GO:0004871), generation of metabolites and energy (GO:0006091), and electron transport chain (GO:0022900). Therefore, a wide range of genes with diverse functions were uniquely differentially regulated by the salt + melatonin treatment.

### The Expression of a Unique Set of Genes Was Highly Induced Exclusively in the Salt-Stressed 02428 Seedlings Treated With Melatonin

We identified a set of genes with up-regulated expression exclusively in exogenous melatonin-treated rice seedlings under salt stress conditions, including genes encoding 30 TFs and 23 phytohormone- or stress-responsive proteins (Table 2). Thirty identified TF genes included 11 AP2/EREBP family members (*OsERF53*, *OsERF62*, *OsERF71*, *OsERF59*, *OsERF124*, *OsCRL5*, and other AP2 genes), 14 Homeobox (HB) TFs (*OSH1*, *OSH3*, *OSH6*, *OSH10*, *OSH15*, *OSH71*, *OSHB4*, *OsHOX8*, *OsHOX14*, *OsHOX21*, *OsHOX24*, *SH5*, and *ROC7*), 3 WRKY TFs (*OsWRKY36*, *OsWRKY66*, and *OsWRKY79*), and OsHsfA7 (Table 2). The up-regulated expression of these TF family’s genes only in response to the salt + melatonin treatment implies these TFs may be involved in the melatonin-mediated transcriptional regulation of salt stress responses in rice.

**TABLE 2:** Genes with up-regulated expression levels exclusively in the 02428 seedlings under salt + melatonin treatment relative to control condition.

Gene name	Log_2_FC	FDR	Function annotation
**Transcription regulation**			
Os01g0224100	2.616	0.00039587	*OsERF53*
Os02g0764700	1.635	0.00067469	*OsERF103*
Os03g0183000	2.749	0.00097458	*OsERF62, OsLG3*
Os03g0232200	8.919	1.79E-12	*OsERF137*
Os03g0770700	5.614	1.15E-08	*OsPLT7*
Os04g0653600	6.215	1.97E-10	*OsPLT1*
Os06g0194000	1.191	0.00305225	*OsERF71*
Os06g0657500	8.936	4.71E-11	*OsPLT2*
Os07g0124700	7.165	4.94E-13	*OsCRL5, AP2/EREBP TF*
Os10g0390800	5.465	0.00059738	*OsERF59*
Os12g0168100	3.722	0.00951772	*OsERF124*
Os03g0727000	4.167	7.91E-21	*OSH1*
Os03g0673000	1.958	0.00222027	*OSH10*
Os07g0129700	5.246	5.92E-14	*OSH15*
Os03g0727200	5.631	0.00019368	*OSH3*
Os01g0302500	4.782	3.50E-09	*OsH6*
Os05g0129700	6.787	3.23E-20	*OSH71*
Os03g0640800	1.151	0.00362214	*OSHB4*
Os07g0581700	6.204	1.84E-09	*OsHOX14*
Os03g0170600	6.653	1.94E-14	*OsHOX21*
Os02g0649300	4.678	1.45E-07	*OsHOX24*
Os10g0377300	2.906	3.02E-06	*OsHOX8*
Os05g0455200	1.609	0.00076726	*SH5*, Homeobox TF
Os06g0562300	4.631	7.99E-08	Homeobox TF
Os08g0136100	1.518	0.00297554	Homeobox TF, ROC7
Os04g0545000	2.599	0.001123	*OsWRKY36*
Os02g0698800	4.877	2.20E-11	*OsWRKY66*
Os01g0182700	2.723	0.00067466	*OsWRKY79*
Os01g0571300	5.237	2.74E-09	*OsHsfA7*
Os02g0598200	2.039	0.00794651	B3 domain TF
**Phytohormone responsive**			
Os04g0673300	1.356	0.00538601	*OsRR6*, cytokinin responsive
Os05g0523300	1.551	0.00916964	*OsIAA18*, auxin responsive
Os02g0141100	1.615	3.04E-05	*OsARF5*, auxin responsive
Os02g0164900	1.106	0.00506874	*OsARF6*, auxin responsive
Os01g0785400	4.791	1.07E-10	*OsGH3-1*, auxin responsive
Os05g0500900	4.365	2.06E-07	*OsGH3-4*, auxin responsive
Os02g0512000	3.041	0.00239214	*OsSAUR10*, auxin responsive
Os03g0347700	8.157	6.99E-12	*BTBN6*, auxin responsive
Os01g0719000	2.555	0.00051309	*DUF581*
Os05g0245300	3.580	1.50E-06	*OsBLE3*, BR responsive
Os03g0602300	1.784	0.00277323	*OsDWARF*, BR responsive
Os12g0586100	2.887	0.00037074	*OsSAPK9*, ABA responsive
Os04g0432000	2.284	0.00518292	*OsSAPK7*, ABA responsive
Os05g0349800	5.638	3.49E-11	*OsEM1*, ABA responsive
**Abiotic stress responsive**			
Os02g0669100	3.206	0.00115264	*OsLEA23*
Os11g0451700	4.731	4.08E-05	*OsLEA25*
Os11g0453900	7.080	5.08E-08	*OsLEA26*
Os11g0454000	5.317	5.38E-05	*OsLEA27*
Os11g0454200	7.152	8.00E-06	*OsLEA28*
Os11g0454300	6.625	1.71E-06	*OsLEA29*
Os05g0214900	1.616	0.00362214	*OsTBL48*
Os11g0547000	2.845	0.00279553	*OsFKF1*
Os03g0432100	2.546	1.25E-08	*OsPPDKA*

Phytohormones are crucial for plant responses to environmental stresses (Verma et al., 2016). The exogenous melatonin treatment of the 02428 seedlings under salt stress conditions increased the expression of genes responsive to different phytohormones, including auxin (*OsIAA18*, *OsARF5*, *OsARF6*, *OsGH3-1*, *OsGH3-4*, and *OsSAUR10*), brassinosteroids (*OsBLE3* and *OsDWARF*), and abscisic acid (ABA) (*OsSAPK7*, *OsSAPK9*, and *OsEM1*). Moreover, the expression of several genes related to abiotic stress responses was exclusively induced by melatonin, including *OsFKF1*, *OsPPDKA*, *OsLEA23*, and five *OsLEA* genes (*OsLEA25*, *OsLEA26*, *OsLEA27*, *OsLEA28*, and *OsLEA29*) clustered on chromosome 11 (Table 2).

### Global Metabolite Changes in the 02428 Seedlings After the Salt and Salt + Melatonin Treatments

**FIGURE 4:**
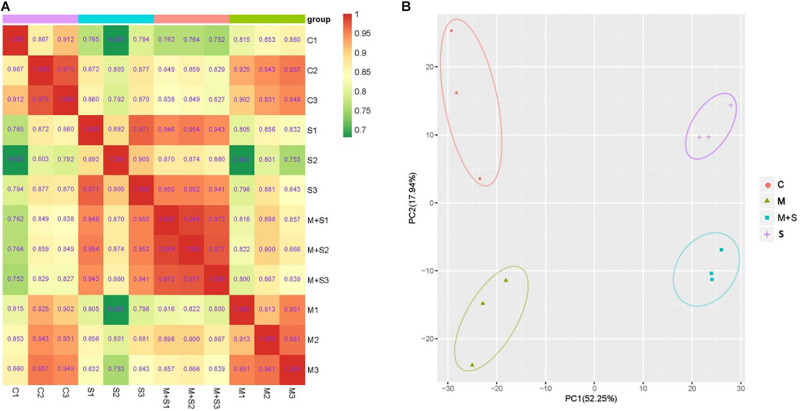
Overall analysis of the metabolite profiling data. **(A)** Pearson correlation coefficients among the samples. **(B)** Principal component analysis of the samples. The x-axis represents the first principal component and the y-axis represents the second principal component. C, control; M, melatonin treatment; S, salt treatment; M + S, salt + melatonin treatment.

To assess the overall metabolic changes due to the exogenous melatonin treatment of the 02428 seedlings exposed to salt stress, we performed a comparative metabolite profiling analysis of 02428 leaf samples following the control, melatonin, salt, and salt + melatonin treatments. A total of 632 metabolites were detected in all samples, of which 308 were significantly differentially abundant in at least one comparison group of melatonin vs control, salt vs control, salt + melatonin vs control, or salt + melatonin vs melatonin (Supplementary Table 4). The results of Pearson’s correlation analysis reflected the reproducibility among the biological samples (Figure 4A), whereas the PCA was used to determine the contribution of the first two primary components (70.19%). The materials for the four treatments were clearly separated into distinct clusters (Figure 4B). These findings confirmed that the reproducibility among the biological samples was sufficient for further analyses.

An examination of the effects of the application of exogenous melatonin revealed 12 metabolites that were differentially abundant between the treated and control samples (Supplementary Table 5). This was consistent with the fact that the expression of only a few genes in the 02428 seedlings was affected by the melatonin treatment. Exogenous melatonin significantly increased the gallic acid, N^1^-acetyl-*N*^2^-formyl-5-methoxykynurenamine (AFMK), syringaldehyde, isoquercitrin, 6-hydroxymusizin-8-*O*-B-D-glucoside, and endogenous melatonin contents. These metabolites reportedly function as antioxidants (Maharaj et al., 2002; Kim, 2007; Hobbs et al., 2018; Shahzad et al., 2018), indicating their possible roles during salt stress responses.

**FIGURE 5:**
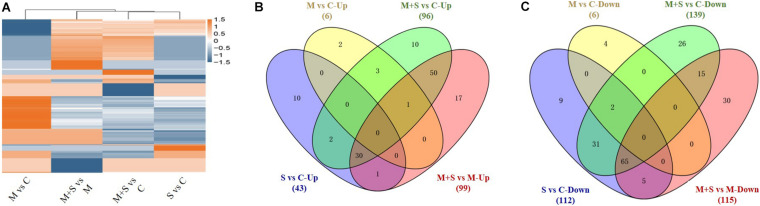
Cluster analysis of all differentially expressed metabolites in 02428 seedlings under different treatments **(A)** and Venn diagram analysis of metabolites with significantly increased **(B)** and decreased **(C)** abundances in the 02428 seedling leaves under different treatments. M, melatonin treatment; M+S, melatonin + salt treatment; S, salt treatment; Up, up-regulated metabolites; Down, down-regulated metabolites.

The abundance of 43/96 and 112/139 metabolites increased and decreased, respectively, in the 02428 seedlings following the salt/salt + melatonin treatments, relative to the control levels (Figure 5 and Supplementary Tables 6, 7). These differentially abundant metabolites under both conditions were analyzed in a Venn diagram, which indicated that the contents of 64 and 41 metabolites increased and decreased, respectively, only in the 02428 seedlings after the salt + melatonin treatment (Figures 5B,C). Thus, the application of exogenous melatonin induced distinct changes to the metabolome of the 02428 seedlings exposed to salt stress. Of these metabolites, the abundances of various amino acids (valine, arginine, aspartic acid, phenylalanine, lysine, and glutamine), organic acids (gallic acid, anchoic acid, hydroxyisobutyric acid, 5-aminovaleric acid, and 4-acetamidobutyric acid), nucleotides (cytosine, pseudouridine, deoxyguanosine, deoxycytidine, deoxyadenosine, and 2-dimethylamino-guanosine), and many secondary metabolites clearly increased in response to exogenous melatonin (Supplementary Table 8).

To examine the main metabolic pathways related to the 02428 seedling responses to exogenous melatonin under salt stress conditions, we assigned the differentially abundant metabolites to KEGG biological pathways. The 64 metabolites with increased abundance only following the salt + melatonin treatment were assigned to 10 KEGG pathways (Supplementary Table 9), including metabolic pathways (ko01100), pyrimidine metabolism (ko00240), phenylalanine metabolism (ko00360), biosynthesis of secondary metabolites (ko01110), and biosynthesis of amino acids (ko01230). In contrast, the 41 metabolites with decreased abundance only after the salt + melatonin treatment were assigned to seven KEGG pathways, including flavone and flavonol biosynthesis (ko00944), fatty acid biosynthesis (ko00061), flavonoid biosynthesis (ko00941), isoflavonoid biosynthesis (ko00943), and tyrosine metabolism (ko00350) (Supplementary Table 10).

### Analysis of the Association Between the Transcriptome and Metabolome Data

**FIGURE 6:**
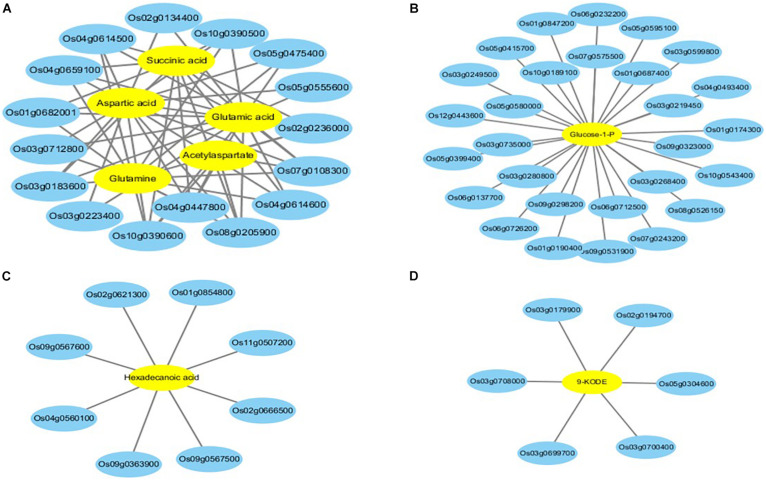
Gene–metabolite correlation network representing the genes and metabolites co-enriched in KEGG pathways involved in melatonin-mediated salt tolerance. **(A)** pathway of alanine, aspartate and glutamate metabolism; **(B)** pathway of amino sugar and nucleotide sugar metabolism; **(C)** pathway of Cutin, suberine and wax biosynthesis; **(D)** pathway of linoleic acid metabolism. The gene–metabolite pairs are connected within the network by edges. Blue and yellow nodes indicate genes and metabolites, respectively.

To determine the relationships between the genes and metabolites related to melatonin-mediated salt stress tolerance, the differentially regulated genes and differentially abundant metabolites in the two comparison groups (i.e., salt treatment vs control and salt + melatonin vs control treatment) were simultaneously assigned to KEGG pathways (*p* < 0.05). A total of 62 and 64 enriched KEGG pathways were identified for the salt stress and salt + melatonin treatments, respectively (Supplementary Figure 3). Of these pathways, alanine, aspartate and glutamate metabolism (ko00250), amino sugar and nucleotide sugar metabolism (ko00250), cutin, suberine and wax biosynthesis (ko00073), and linoleic acid metabolism (ko00591) were significantly enriched only for the salt + melatonin treatment. The metabolites involved in these four pathways are listed in Table 3. The transcriptome and metabolome data were also compared by a Pearson correlation analysis. Gene–metabolite correlation networks were constructed for these four enriched pathways (Figure 6 and Supplementary Table 11). Five genes encoding lipoxygenases (LOXs) and one gene encoding phospholipase A2 (PLA2) were highly positively or negatively correlated with 9-oxooctadeca-10, 12-dienoic acid (9-KODE), which is involved in linoleic acid metabolism. Three genes encoding cytochrome P450s, two genes encoding NAD-dependent epimerases, and three genes encoding GMC oxidoreductase, fatty acid hydroxylase, and transferase were negatively correlated with hexadecenoic acid, which is related to cutin, suberin, and wax biosynthesis. Additionally, 22 genes involved in amino sugar and nucleotide metabolism were correlated with glucose-1-phosphate, whereas 13 genes [encoding nine aminotransferases (ATs), glutamine amidotransferase (GAT), FAD-dependent oxidoreductase (FOXRED), glutamine synthetase (GS), and pyridine nucleotide-disulfide oxidoreductase (PYROX)] involved in amino acid metabolism were correlated with succinic acid, L-aspartic acid, glutamic acid, L-glutamine, and *N*-acetylaspartate (Supplementary Table 11). These results suggest that the differentially regulated genes related to the metabolism of amino acids, sugars, and linoleic acid and wax biosynthesis were highly correlated with the corresponding metabolites, indicative of their importance for melatonin-mediated salt stress responses in rice.

**TABLE 3:** Co-mapped molecular pathways based on an integrated comparison of metabolite and transcript profiling data for 02428 seedlings under salt + melatonin treatment relative to control condition.

Pathway	Metabolite ID	Metabolite name	Log2 (FC)
	mws0192	Succinic acid	−0.81
	mws0219	L-Aspartic Acid	−2.10
Alanine, aspartate, and glutamate	mws1706	Glutamic acid	−1.01
metabolism (ko00250)	pme0193	L-Glutamine	0.79
	pme2559	*N*-Acetylaspartate	1.29
Amino sugar and nucleotide sugar metabolism (ko00520)	mws1090	Glucose-1-phosphate	−1.43
Cutin, suberine, and wax biosynthesis (ko00073)	pmn001578	Hexadecanoic acid	−0.83
Linoleic acid metabolism (ko00591)	pmb2787	9-KODE	2.48

## Discussion

In plants, melatonin is a relatively recently discovered phytohormone with multiple biological functions. It stimulates plant growth and development and mediates adaptive responses to biotic and abiotic stresses (Sun et al., 2020b). In the present study, rice seedlings were treated with exogenous melatonin to improve salt tolerance. The results of various analyses indicated that melatonin-treated seedlings grew better and were more able to maintain relatively low sodium contents under salt stress conditions than the untreated seedlings. Accordingly, melatonin-mediated salt tolerance may be associated with ion homeostasis. This is consistent with the results of a previous study, in which melatonin improved rice salt tolerance by modulating potassium homeostasis (Liu et al., 2020). In the current study, the detrimental effects of salt stress, including decreased chlorophyll contents and increased MDA contents, in 02428 seedlings under saline conditions were mitigated by the application of exogenous melatonin. These findings provide evidence that melatonin can function as a general stress regulator that maintains ion homeostasis, prevents membrane lipid oxidation, and enhances photosynthetic activities (Wang et al., 2018).

A global gene expression analysis conducted to investigate the molecular basis of melatonin-mediated salt stress tolerance revealed that an exogenous melatonin treatment only slightly affects gene expression in 02428 seedlings under control conditions. Specifically, 16 genes, including *OsEXPB2*, *OsHsp40*, and *OsBBX20*, were differentially expressed. Earlier functional analyses proved that OsEXPB2, OsHsp40, and OsBBX20 are involved in cell wall loosening, stress responses, and growth regulation, respectively (Huang et al., 2012; Zou et al., 2015; Wang et al., 2019). Thus, melatonin has diverse functions related to plant growth and development. The relatively limited change in global gene expression is consistent with the lack of observable phenotypic differences between the control and melatonin-treated 02428 seedlings.

Melatonin reportedly has multiple functions related to plant responses to abiotic stresses (Liang et al., 2015; Wang et al., 2018; Liu et al., 2020). In the present study, the application of exogenous melatonin alleviated the adverse effects of salt stress on the salt-sensitive 02428 seedlings, reflecting the activation of melatonin-mediated systems related to salt tolerance. A GO enrichment analysis of the genes with up- or down-regulated expression levels exclusively in response to the salt + melatonin treatment revealed that exogenous melatonin substantially induces the expression of genes involved in developmental processes, hormone and abiotic stress responses, transcriptional regulation, and carbohydrate metabolic processes (Supplementary Figure 2), implying multiple molecular pathways contribute to the melatonin-mediated salt tolerance of rice.

Transcription factors are critical regulators of plant development and essential plant activities such as responses to environmental stresses and hormones (Baillo et al., 2019). Previous studies identified several genes encoding TFs, including DREB/CBF, WRKY, and MYB TFs, involved in melatonin-mediated stress signaling pathways in plants (Shi et al., 2015a, 2016; Wang et al., 2018). In this study, the expression levels of a unique set of TF genes, including 11 AP2/EREBP, 14 HB, and 3 WRKY genes, were significantly up-regulated in the salt-stressed seedlings treated with exogenous melatonin, implying that melatonin-mediated salt tolerance might partially rely on the AP2/EREBP–HB–WRKY transcriptional cascade. Some of these 11 AP2/EREBP TFs, including OsERF103, OsERF62, and OsERF71, are reportedly important for abiotic stress tolerance (Dietz et al., 2010; Lee et al., 2016; Xiong et al., 2018). Moreover, several of the identified HB TFs, such as OSH1, OSH15, OSH6, and OsHOX24, were previously revealed to affect growth and development as well as responses to environmental stimuli (Sato et al., 1996; Park et al., 2007; Bhattacharjee et al., 2017; Yoon et al., 2017). Whereas the WRKY proteins are important regulators of various physiological processes, including pathogen defense, senescence, and trichome development (Eulgem et al., 2000). The melatonin-mediated up-regulated expression of the genes encoding these TFs in rice seedlings under saline conditions suggests that the salt-induced inhibition of growth and development was offset and stress-responsive downstream cascades were activated by the exogenous melatonin. Notably, a few of the AP2/EREBP and HB family TFs were previously reported to participate in the biosynthesis of endogenous melatonin in plants (Wei et al., 2018) and in animals (Rohde et al., 2014, 2019). Whether these two types of TFs are related to melatonin biosynthesis in rice plants exposed to salt stress remains to be determined.

Melatonin is crucial for the modulation of gene expression related to plant hormones (Arnao and Hernández-Ruiz, 2018). The genes identified in this study with up-regulated expression levels specifically in rice seedlings that underwent the salt + melatonin treatment are involved in phytohormone responses. These genes included the auxin-responsive genes *OsIAA8*, *OsARF5*, *OsARF6*, *OsGH3-1*, *OsGH3-2*, and *OsSAUR10* (Jain et al., 2006; Wang et al., 2007; Song et al., 2009; Fu et al., 2011; Kong et al., 2019), the ABA-responsive genes *OsSAPK7*, *OsSAPK9*, and *OsEM1* (Xu et al., 2013; Yu et al., 2016), and the brassinosteroid-responsive genes *OsBLE3* and *OsDWARF* (Hong et al., 2002; Yang et al., 2006). Therefore, exogenous melatonin may activate auxin/ABA/brassinosteroid signaling pathways. A recent study confirmed that melatonin responses in rice are closely associated with the auxin signaling pathway (Liang et al., 2017). Additionally, ABA may serve as a downstream signal of melatonin during responses to abiotic stresses (Banerjee and Roychoudhury, 2019). Hence, melatonin and phytohormones may synergistically function as key regulators of plant stress responses.

Our comparative metabolite profiling revealed melatonin induced distinct changes to metabolite contents in the 02428 seedlings. The abundances of a few metabolites, including gallic acid, syringaldehyde, AFMK, and isoquercitrin, increased substantially following the exogenous melatonin treatment of the 02428 seedlings under control conditions. These metabolites were previously reported to function as antioxidants (Maharaj et al., 2002; Kim, 2007; Hobbs et al., 2018; Shahzad et al., 2018), reflecting their intrinsic role in melatonin-mediated stress responses. However, the salt + melatonin treatment affected the metabolite contents more than the salt treatment alone. Specifically, 64 metabolites were more abundant in the melatonin-treated salt-stressed rice plants than in the salt-stressed plants (Supplementary Table 9). Six of these metabolites (gallic acid, diosmetin, cyanidin 3-*O*-galactoside, AFMK, 5-hydroxy-L-tryptophan, and melatonin) function as important antioxidants in response to environmental stresses (Tan et al., 2001; Kim, 2007; Bazghaleh et al., 2018; Yang et al., 2018; Vigliante et al., 2019), suggesting that melatonin activates multiple antioxidant pathways to protect seedlings from damages due to salt stress. Moreover, other metabolites, such as amino acids, organic acids, nucleotides, and their derivatives, were highly accumulated in the seedlings that underwent the salt + melatonin treatment, suggestive of their beneficial physiological roles during melatonin-mediated responses to salt stress. Physiologically, many of these metabolites may function as osmolytes in the cytoplasm under salt stress conditions (Slama et al., 2015). Alternatively, they may compensate for ionic imbalances and contribute to nitrogen/carbon metabolism (Parida and Das, 2005). These results indicate that the melatonin-induced accumulation of these metabolites might directly lead to the improved salt tolerance of the 02428 seedlings.

Combining transcriptome and metabolome analyses enabled a more comprehensive investigation of the molecular processes associated with gene–metabolite networks related to plant responses to abiotic stresses (Kaplan et al., 2007). As the end products of gene expression, metabolites serve as a direct link between the genotype and phenotype, implying metabolite profiling data may be useful for elucidating the molecular basis of melatonin-mediated salt tolerance. In the present study, several gene–metabolite correlations were identified in the 02428 seedlings treated with melatonin under salt stress conditions. For example, six genes (five *LOX* genes and *PLA2*) were correlated with 9-KODE, which is related to linoleic acid metabolism. Furthermore, 9-KODE, which is produced in a reaction catalyzed by LOX, is an intermediate in the jasmonic acid biosynthesis pathway (Babenko et al., 2017; Nishiguchi et al., 2018), implying the jasmonic acid signaling pathway is involved in the melatonin-mediated salt stress response. Moreover, 13 genes (encoding nine ATs, GAT, GS, FOXRED, and PYROX) related to amino acid metabolism were highly correlated with succinic acid and three amino acids (L-aspartic acid, glutamic acid, and L-glutamine). Both GAT and GS are involved in arginine biosynthesis (Holden et al., 1998), L-glutamine biosynthesis, and nitrogen cycling (Suzuki and Knaff, 2005; Yang et al., 2016). A previous study indicated that ATs are important for amino acid biosynthesis and catabolism as well as carbon and nitrogen shuttles (Liepman and Olsen, 2004). This enriched gene–metabolite network provides further evidence that amino acid metabolism is a major part of the mechanism underlying the melatonin-mediated salt tolerance of rice. Notably, metabolites such as succinic acid, L-aspartic acid, glutamic acid, glucose-1-phosphate were decreased in 02428 seedlings under melatonin + salt treatment relative to control (Table 3), these decreased metabolites are energy-associated metabolites, which are essential for plant growth and response to various stresses (Less et al., 2010; Ludewig and Flügge, 2013), implying melatonin could activate energy-saving program to response stress by inhibiting the biosynthesis of metabolites. However, further experiments are required to confirm the melatonin-mediated gene-metabolite networks of rice response to salt stress.

## Conclusion

In conclusion, this study provided useful evidence of the protective effect of exogenous melatonin in rice seedlings response to salt stress. Exogenous melatonin could enhance the salt tolerance of rice plants by activating unique TFs transcriptional cascade, multiple antioxidant pathways, and modulation of metabolite homeostasis.

## Data Availability Statement

The datasets presented in this study can be found in online repositories. The names of the repository/repositories and accession number(s) can be found below: The raw sequence data have been deposited in the Genome Sequence Archive in National Genomics Data Center, Beijing Institute of Genomics, Chinese Academy of Sciences, under accession number CRA003581 that are publicly accessible at https://bigd.big.ac.cn/gsa. The metabolite data have been deposited in the National Genomics Data Center, Beijing Institute of Genomics, Chinese Academy of Sciences, under accession number OMIX220 that are publicly accessible at: https://bigd.big.ac.cn/omix.

## Author Contributions

ZX and JW performed the experiments. ZX, WW, and YW analyzed RNA-Seq and metabolite data. JX and ZL provided assistance on the phenotyping experiment. BF and XZ designed the experiments and wrote the manuscript. All authors contributed to the article and approved the submitted version.

## Conflict of Interest

The authors declare that the research was conducted in the absence of any commercial or financial relationships that could be construed as a potential conflict of interest.

## Footnotes

1 https://bigd.big.ac.cn/gsa

2 http://bioinfo.cau.edu.cn/agriGO/

3 https://bigd.big.ac.cn/omix

4 http://systemsbiology.cau.edu.cn/agriGOv2/index.php

## Supplementary Material

Supplementary Figure 1 Validation of the expression of 11 genes related to melatonin biosynthesis and catabolism by RT-qPCR. **(A)** RT-qPCR analysis of the genes involved in melatonin biosynthesis and catabolism. The genes and the corresponding PCR primers are listed in Supplementary Table 1. Rice seedlings were maintained under control **(C)** conditions or treated with 100 mM salt (S), 10 μM melatonin (M), or 100 mM salt + 10 μM melatonin (M+S). ^∗^, ^∗∗^, and ^∗∗∗^ indicate significant differences between the treated and control samples at *p* < 0.05, *p* < 0.01, and *p* < 0.001, respectively. **(B)** Correlation analysis revealing the consistency between the RNA sequencing and RT-qPCR data. The RNA sequencing data (x-axis) were plotted against the RT-qPCR data (y-axis).

Supplementary Figure 2 Gene Ontology (GO) enrichment analysis of the genes with up- and down-regulated expression levels exclusively in the 02428 seedlings that underwent the salt + melatonin treatment. The x-axis presents the GO function classifications and the y-axis presents the number of differentially expressed genes.

Supplementary Figure 3 Analyses of the association between the genes and metabolites related to melatonin-mediated salt stress tolerance. The differentially regulated genes and differentially abundant metabolites in the two comparison groups (control vs salt treatment and control vs salt + melatonin treatment) were simultaneously mapped to KEGG pathways (*p* < 0.05). **(A)** Enriched pathways for the salt + melatonin treatment. **(B)** Enriched pathways for the salt treatment.

Supplementary Table 1 Genes and the corresponding primers for the quantitative real-time PCR analysis.

Supplementary Table 2 Genes differentially expressed in the 02428 seedlings under salt stress conditions.

Supplementary Table 3 Genes differentially expressed in the 02428 seedlings after the salt + melatonin treatment.

Supplementary Table 4 Metabolites detected in the 02428 seedlings after different treatments.

Supplementary Table 5 Differentially abundant metabolites in the 02428 seedlings after the exogenous melatonin treatment.

Supplementary Table 6 The differentially abundant metabolites in 02428 seedlings under salt stress.

Supplementary Table 7 The differentially abundant metabolites in 02428 seedling under salt plus melatonin treatment.

Supplementary Table 8 Sixty-four metabolites exclusively increased in 02428 seedling under salt and melatonin treatment.

Supplementary Table 9 KEGG annotation of the exclusively increased metabolites in 02428 seedling under salt + melatonin treatment relative to control.

Supplementary Table 10 KEGG annotation of the exclusively decreased metabolites in 02428 seedling under salt + melatonin treatment relative to control.

Supplementary Table 11 Gene-metabolite correlation networks identified by Pearson’s correlation analysis.
