# Supplementary material for: Integrated Analysis of the Transcriptome and Metabolome Revealed the Molecular Mechanisms Underlying the Enhanced Salt Tolerance of Rice Due to the Application of Exogenous Melatonin
Source: Front Plant Sci. 2021 Jan 14;11:618680. doi: 10.3389/fpls.2020.618680 (PMC7840565; doi:10.3389/fpls.2020.618680)
Supplement: Supplementary Figure 1 — Validation of the expression of 11 genes related to melatonin biosynthesis and catabolism by RT-qPCR. (A) RT-qPCR analysis of the genes involved in melatonin biosynthesis and catabolism. The genes and the corresponding PCR primers are listed in Supplementary Table 1. Rice seedlings were maintained under control (C) conditions or treated with 100 mM salt (S), 10 μM melatonin (M), or 100 mM salt + 10 μM melatonin (M+S). ∗, ∗∗, and ∗∗∗ indicate significant differences between the treated and control samples at p < 0.05, p < 0.01, and p < 0.001, respectively. (B) Correlation analysis revealing the consistency between the RNA sequencing and RT-qPCR data. The RNA sequencing data (x-axis) were plotted against the RT-qPCR data (y-axis). [file Presentation_1.PPTX]

## Slide 1
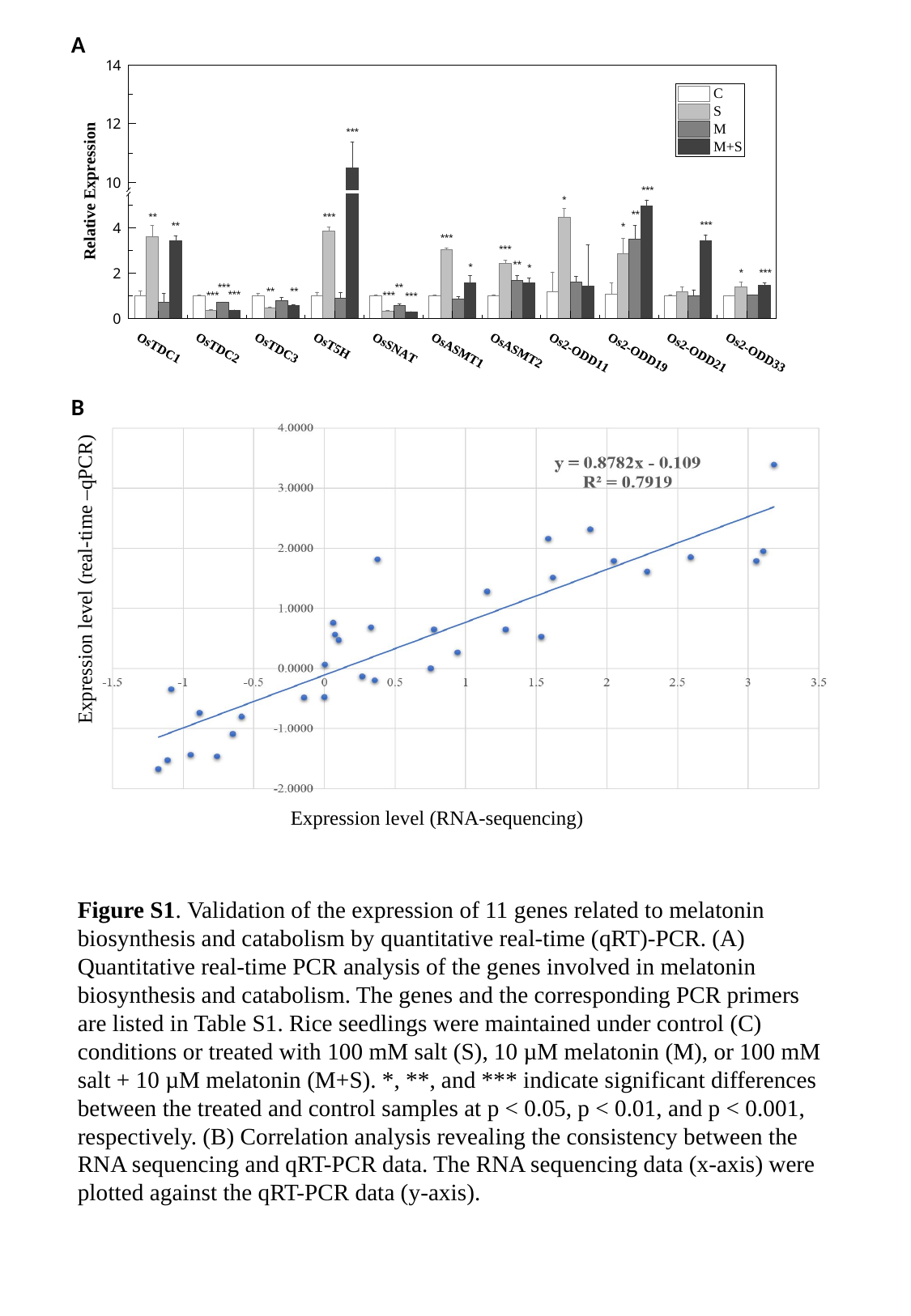

A
B
Expression level (real-time –qPCR)
Expression level (RNA-sequencing)
Figure S1. Validation of the expression of 11 genes related to melatonin biosynthesis and catabolism by quantitative real-time (qRT)-PCR. (A) Quantitative real-time PCR analysis of the genes involved in melatonin biosynthesis and catabolism. The genes and the corresponding PCR primers are listed in Table S1. Rice seedlings were maintained under control (C) conditions or treated with 100 mM salt (S), 10 µM melatonin (M), or 100 mM salt + 10 µM melatonin (M+S). *, **, and *** indicate significant differences between the treated and control samples at p < 0.05, p < 0.01, and p < 0.001, respectively. (B) Correlation analysis revealing the consistency between the RNA sequencing and qRT-PCR data. The RNA sequencing data (x-axis) were plotted against the qRT-PCR data (y-axis).
