# Supplementary material for: Integrated Analysis of the Transcriptome and Metabolome Revealed the Molecular Mechanisms Underlying the Enhanced Salt Tolerance of Rice Due to the Application of Exogenous Melatonin
Source: Front Plant Sci. 2021 Jan 14;11:618680. doi: 10.3389/fpls.2020.618680 (PMC7840565; doi:10.3389/fpls.2020.618680)
Supplement: Supplementary Figure 2 — Gene Ontology (GO) enrichment analysis of the genes with up- and down-regulated expression levels exclusively in the 02428 seedlings that underwent the salt + melatonin treatment. The x-axis presents the GO function classifications and the y-axis presents the number of differentially expressed genes. [file Presentation_2.PPTX]

## Slide 1
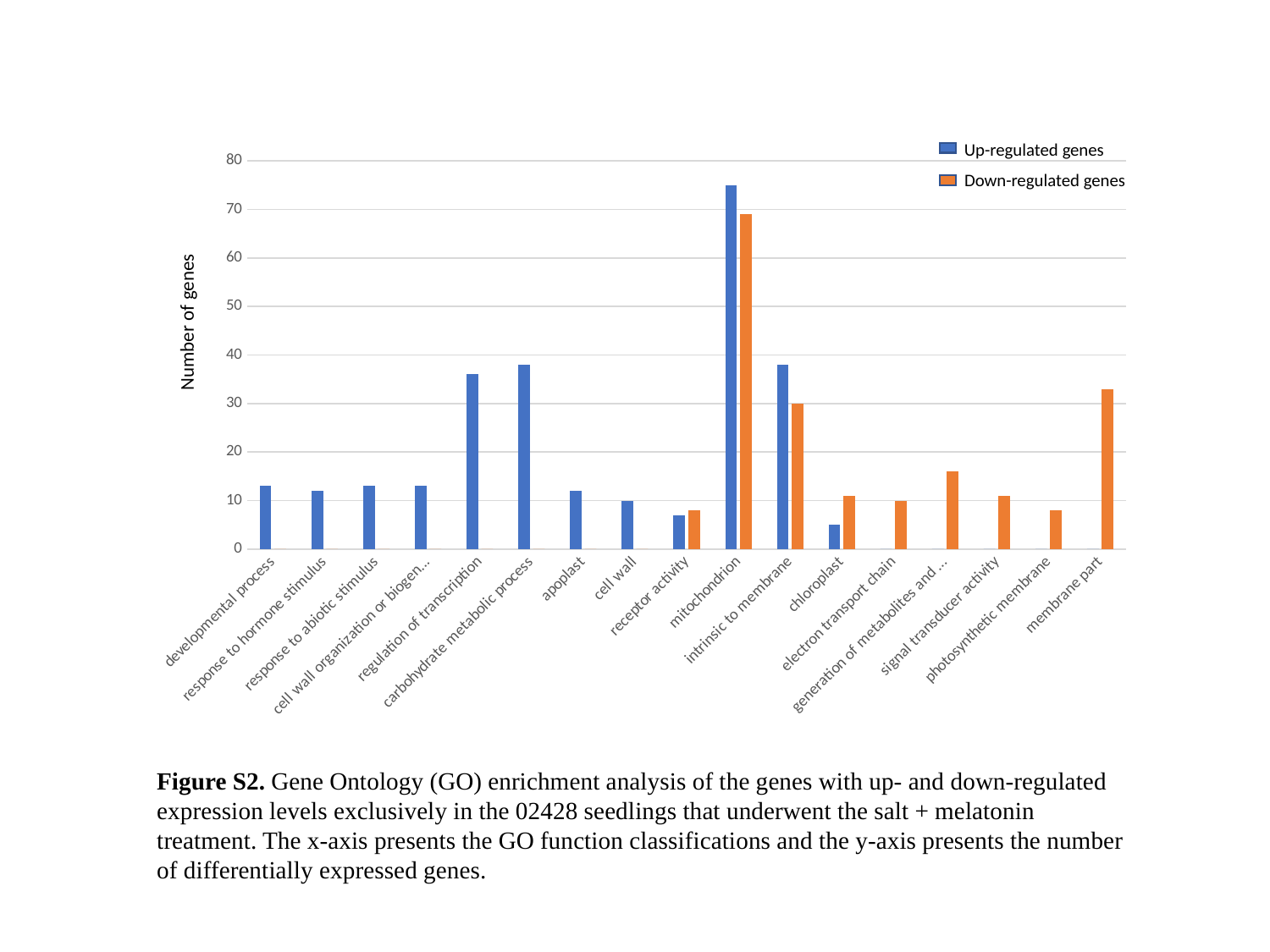

### Chart
| Category | | |
|---|---|---|
| developmental process | 13.0 | 0.0 |
| response to hormone stimulus | 12.0 | 0.0 |
| response to abiotic stimulus | 13.0 | 0.0 |
| cell wall organization or biogenesis | 13.0 | 0.0 |
| regulation of transcription | 36.0 | 0.0 |
| carbohydrate metabolic process | 38.0 | 0.0 |
| apoplast | 12.0 | 0.0 |
| cell wall | 10.0 | 0.0 |
| receptor activity | 7.0 | 8.0 |
| mitochondrion | 75.0 | 69.0 |
| intrinsic to membrane | 38.0 | 30.0 |
| chloroplast | 5.0 | 11.0 |
| electron transport chain | 0.0 | 10.0 |
| generation of metabolites and energy | 0.0 | 16.0 |
| signal transducer activity | 0.0 | 11.0 |
| photosynthetic membrane | 0.0 | 8.0 |
| membrane part | 0.0 | 33.0 |Up-regulated genes
Down-regulated genes
Number of genes
Figure S2. Gene Ontology (GO) enrichment analysis of the genes with up- and down-regulated expression levels exclusively in the 02428 seedlings that underwent the salt + melatonin treatment. The x-axis presents the GO function classifications and the y-axis presents the number of differentially expressed genes.
