# Supplementary material for: Integrated Analysis of the Transcriptome and Metabolome Revealed the Molecular Mechanisms Underlying the Enhanced Salt Tolerance of Rice Due to the Application of Exogenous Melatonin
Source: Front Plant Sci. 2021 Jan 14;11:618680. doi: 10.3389/fpls.2020.618680 (PMC7840565; doi:10.3389/fpls.2020.618680)
Supplement: Supplementary Figure 3 — Analyses of the association between the genes and metabolites related to melatonin-mediated salt stress tolerance. The differentially regulated genes and differentially abundant metabolites in the two comparison groups (control vs salt treatment and control vs salt + melatonin treatment) were simultaneously mapped to KEGG pathways (p < 0.05). (A) Enriched pathways for the salt + melatonin treatment. (B) Enriched pathways for the salt treatment. [file Presentation_3.PPTX]

## Slide 1
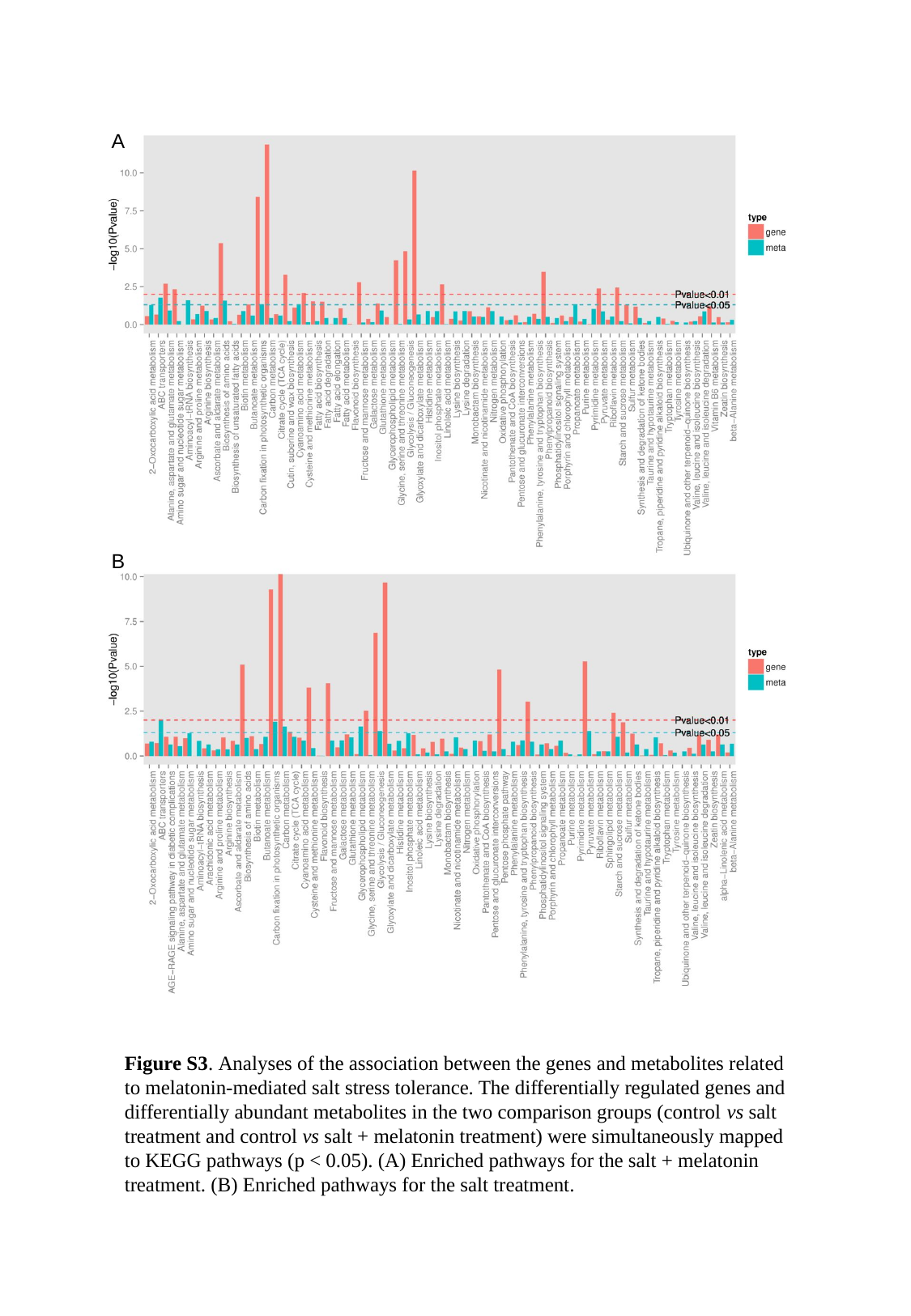

A
B
Figure S3. Analyses of the association between the genes and metabolites related to melatonin-mediated salt stress tolerance. The differentially regulated genes and differentially abundant metabolites in the two comparison groups (control vs salt treatment and control vs salt + melatonin treatment) were simultaneously mapped to KEGG pathways (p < 0.05). (A) Enriched pathways for the salt + melatonin treatment. (B) Enriched pathways for the salt treatment.
