# Supplementary material for: Integrated Analysis of the Transcriptome and Metabolome Revealed the Molecular Mechanisms Underlying the Enhanced Salt Tolerance of Rice Due to the Application of Exogenous Melatonin
Source: Front Plant Sci. 2021 Jan 14;11:618680. doi: 10.3389/fpls.2020.618680 (PMC7840565; doi:10.3389/fpls.2020.618680)
Supplement: Supplementary file 4 [file Presentation_4.PPTX]

## Slide 1
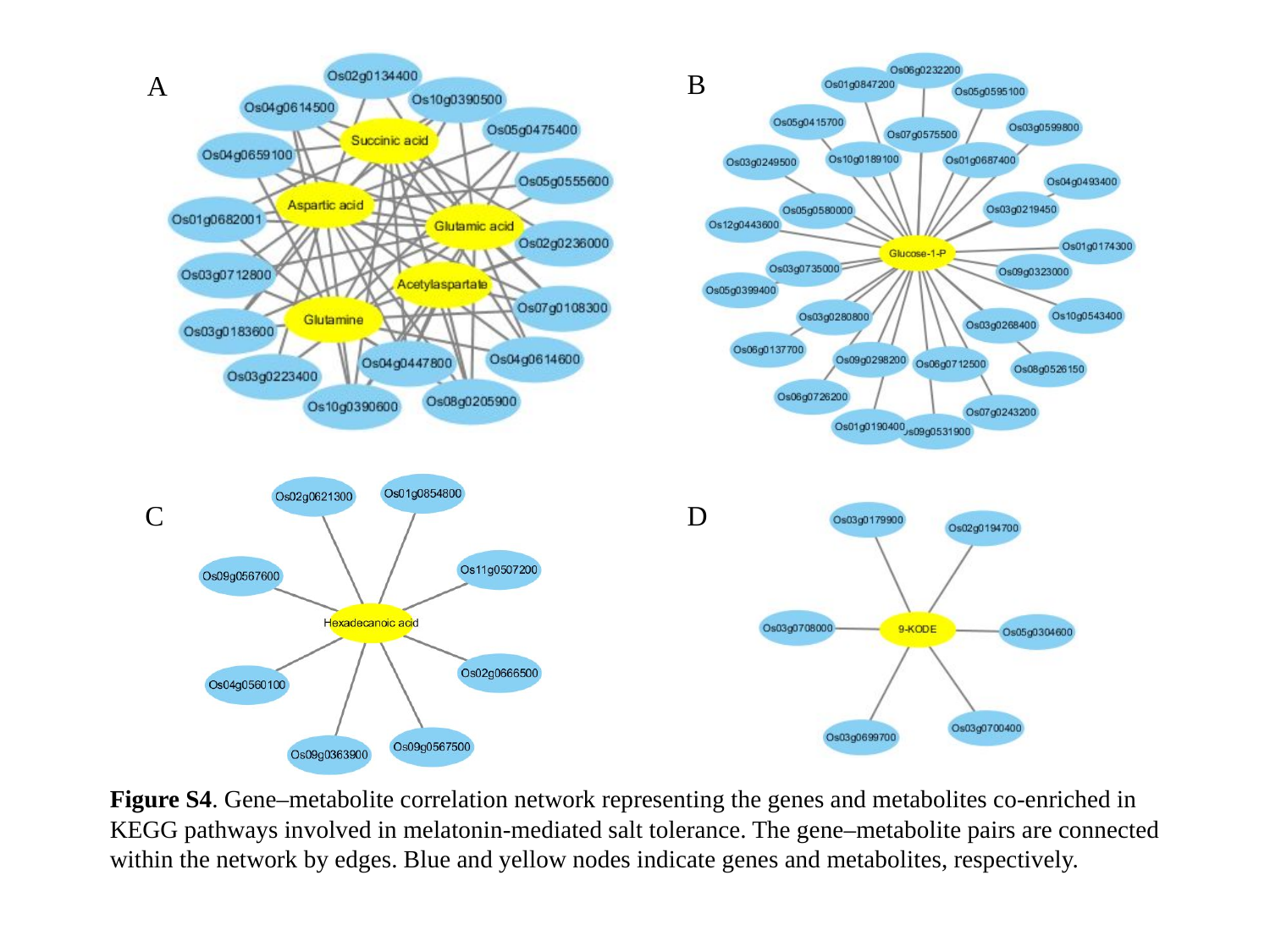

B
A
C
D
Figure S4. Gene–metabolite correlation network representing the genes and metabolites co-enriched in KEGG pathways involved in melatonin-mediated salt tolerance. The gene–metabolite pairs are connected within the network by edges. Blue and yellow nodes indicate genes and metabolites, respectively.
